# D-galactose-induced brain ageing model: A systematic review and meta-analysis on cognitive outcomes and oxidative stress indices

**DOI:** 10.1371/journal.pone.0184122

**Published:** 2017-08-30

**Authors:** Saeed Sadigh-Eteghad, Alireza Majdi, Sarah K. McCann, Javad Mahmoudi, Manouchehr S. Vafaee, Malcolm R. Macleod

**Affiliations:** 1 Neurosciences Research Center (NSRC), Tabriz University of Medical Sciences, Tabriz, Iran; 2 Centre for Clinical Brain Sciences, University of Edinburgh, Edinburgh, Scotland, United Kingdom; 3 Department of Neuroscience and Pharmacology, University of Copenhagen, Copenhagen, Denmark; 4 Department of Clinical Neurosciences, The University of Edinburgh, Edinburgh, United Kingdom; Hokkaido Daigaku, JAPAN

## Abstract

Animal models are commonly used in brain ageing research. Amongst these, models where rodents are exposed to d-galactose are held to recapitulate a number of features of ageing including neurobehavioral and neurochemical changes. However, results from animal studies are often inconsistent. To better understand the characteristics of the model and effects of d-galactose on neurobehavioral and neurochemical outcomes in rodents we performed a systematic review and meta-analysis. We applied random-effects meta-analysis to evaluate the effect of study features. Our results give an overview of the characteristics of the d-galactose rodent ageing model, including neurobehavioral and neurochemical outcomes. We found that few studies took measures to reduce risks of bias, and substantial heterogeneity in the reported effects of d-galactose in included studies. This highlights the need for improvements in the use of the d-galactose rodent ageing model if it is to provide useful in the development of drugs to treat human ageing.

## Introduction

Ageing is a time-dependent multifaceted process in which progressive loss of physiological integrity causes functional impairment and decreased quality of life [1, 2].

One of the cardinal features of ageing is brain ageing, manifest in a wide spectrum of behavioural deficits including anxiety and impaired cognitive function. Changes in brain structural connectivity, decrease in neurogenesis, lipids peroxidation, oxidative stress, mitochondrial dysfunction, decline in neurotransmitters levels and beta amyloid (Aβ) overproduction have all been suggested to be major mediators of brain ageing and age-related neurologic disorders [3–5].

Animal models can be extremely valuable tools for studying biological mechanisms, for testing hypotheses generated from clinical research, and for testing the efficacy of candidate interventions. Such models should have demonstrable reliability, predictive validity, construct validity and relevance if findings from such studies are to translate from bench to bedside [6].

In the last 10 years, ageing research using animals has gained an increasing attention with the availability of drug-induced animal models which can be used to study accelerated ageing. D-galactose-injected rodent models recapitulate many features of brain ageing and have been extensively applied to study the mechanisms of brain ageing [3, 7, 8]. Following administration, d-galactose reacts with … to form advanced glycation end-products (AGEs) and cause oxidative stress. This in turn can lead to increased malondialdehyde (MDA) levels and decreased superoxide dismutase (SOD) and glutathione peroxidase (GSH-px) activities [9–11]. Its administration in rodents also has been reported to cause neurobehavioral changes including cognition and motor impairment; reduced neurogenesis; neurodegeneration; and caspase-dependent apoptosis and mitochondrial dysfunction [12].

However, findings from different laboratories, often from small studies, are inconsistent. Systematic reviews and meta-analysis are techniques to provide an unbiased and transparent summary of existing research [13, 14]. They can be helpful in the design of clinical trials [15, 16] and in understanding discrepancies between the results of preclinical and clinical trials [14]. Here, we report a systematic review and meta-analysis to appraise d-galactose-induced brain ageing as a prevalent ageing model in rodent.

## Methods

The study protocol was defined in advance and is available at www.dcn.ed.ac.uk/camarades/research.html#protocols; further details of the methodology can be found in Vesterinen et al [17].

### Search strategy

We electronically searched two databases (MEDLINE via PubMed and SCOPUS) for studies that used d-galactose as a brain-aging-inducing agent in rodent, using the keywords “Brain”, “aging”, “d-galactose” and “Rodent” as follows: [(brain)] AND [(aging) OR (sensense) OR (geriatric) OR (gerontic)] AND [(rodent) OR (rat) OR (mice) OR (mouse) OR (rattus) or (mus)] AND [d-galactose]. Two investigators used the SyRF platform (app.syrf.org.uk) independently to screen title, abstract and where necessary full text, judging the work against the inclusion and exclusion criteria. Where there are disagreements, the SyRF platform automatically serves the citation to a third investigator for adjudication. There was no date and language restriction in our search and the study was restricted to “other (i.e. non-human) animals”.

### Inclusion and exclusion criteria

We included all rodent (*e*.*g*. mouse and rat) studies reported in full-text publications. Which used d-galactose to induce features of ageing We included any route of administration, dose, and dose timing and frequency. The primary outcome measure was cognition-related neurobehavioral outcome and secondary outcome measures were changes in the abundance of biochemical markers MDA, GSH-px, SOD, protein carbonyl (PC), Caspase-3, Bcl-2, Bax and AChE (acetylcholine esterase).

We excluded studies not using rodents and those using compounds other than d-galactose, and *ex vivo* or *in vitro* (primary culture or cell line) experiments.

### Data extraction

We extracted the author, publication year and type, animal characteristics (including species, strain, and sex, weight and age range or categorical age) and supplier, d-galactose model details (including route, dose and frequency of injection and duration of exposure), study quality evaluation, and the reporting of measures to reduce the risk of bias (listed below). We also recorded data for the nature of the outcome reported (neurobehavioural or neurochemical), the number of d-galactose groups served by the control group, and the number of animals per group, mean outcome and SD or SEM. We did not record the time of outcome assessment.

When a single publication reported more than one experiment, the data were evaluated as independent experiments. Where neurobehavioral or neurochemical outcomes were reported more than once in the same cohort of animals we recorded only data for the last assessment time. For graphically-presented data, we measured values from graphs using Universal Desktop Ruler, version 2.9 or contacted the manuscript authors for more information.

### Methodological quality of studies

The internal validity of the enrolled studies (*e*.*g*. selection, performance, detection and attrition bias) and other study quality measures (*e*.*g*. reporting quality, power) were assessed using a modified version of the CAMARADES' study quality checklist [18] which comprised: publication in peer-reviewed journal, randomization to treatment or control, allocation concealment, blinded assessment of outcome, statement of inclusion and exclusion of animals from the study, sample-size calculation, statement of compliance with regulatory requirements and statement regarding possible conflict of interest.

### Statistical analysis

We expected substantial heterogeneity between studies so used a random effects model. The primary outcome was the overall effect of d-galactose on neurobehavioral outcome. Secondary outcomes were the effect of d-galactose on 8 biochemical outcomes, with a Holm-Bonferroni adjusted critical value for *p* of 0.006. Stratifications were considered in two domains, with 8 aspects of study design and 8 aspects of study quality, each domain tested at *p*<0.05 overall for neurobehaviour, *p*<0.006 for each biochemical outcome, giving critical values for *p* across 8 tests of 0.006 and 0.0008 respectively. For study quality items we also calculated an effect size as the change in effect observed in studies at high risk of bias, along with their 99.5% confidence intervals. Since we used the statistically more conservative standardized mean difference (SMD) we assessed the significance of differences between n groups by partitioning heterogeneity and by using Chi-square test with n-1 degrees of freedom (11). For continuous variables, we divided these into quartiles for partitioning of heterogeneity (STATA, version 10). Due to the limitations of using funnel plotting [16], Egger’s regression [17] and trim and fill in the assessment of SMD publication bias (Wever et al, manuscript under consideration), these tests were not applied to assess publication bias in this literature.

## Results

### General study characteristics

**Fig 1:**
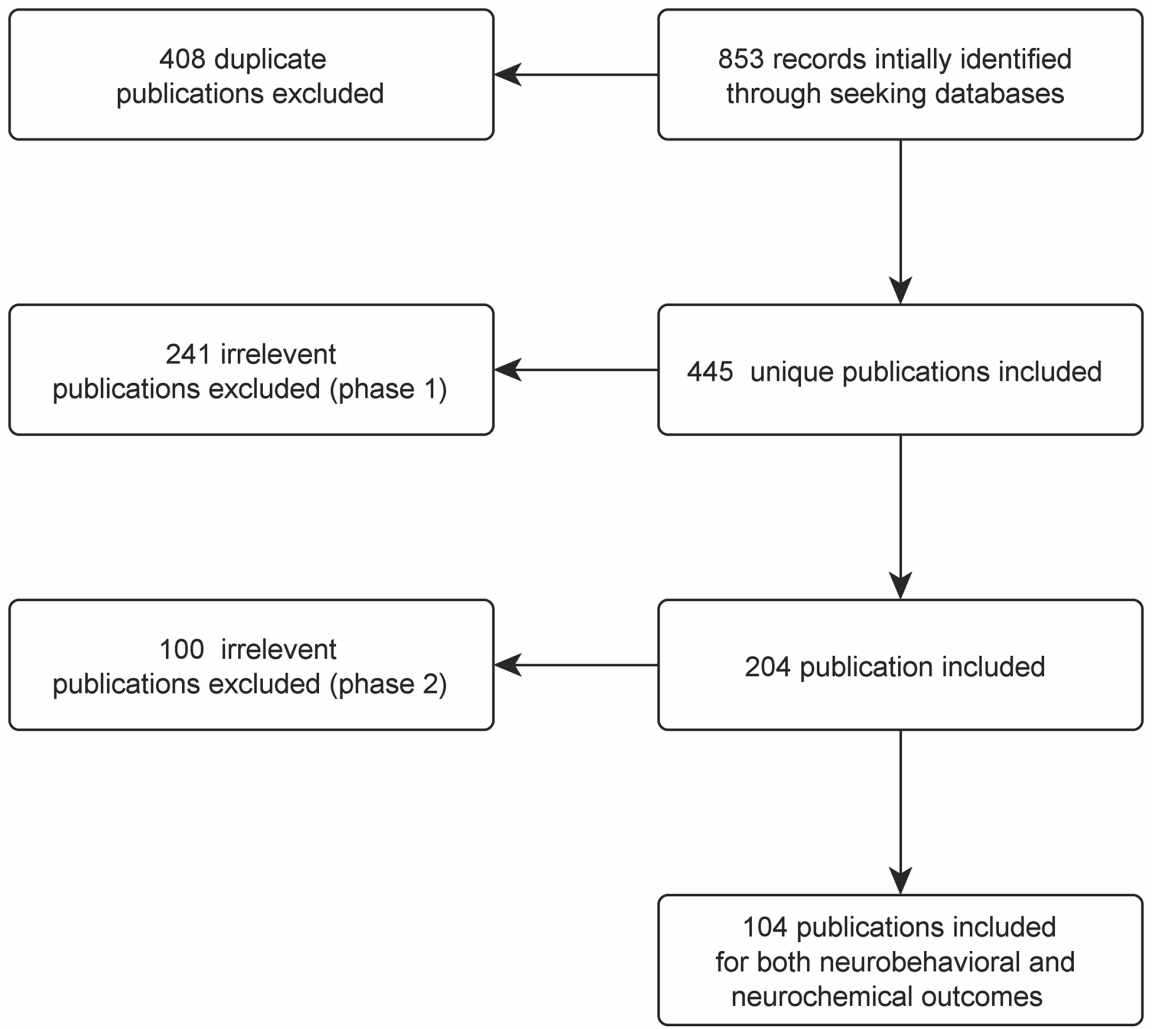
Literature selection summary showing the number of inclusions and exclusions from the initial search. In phase 1, the relevancy of the publications was assessed based on the title and abstract, however, in the phase 2 it was evaluated based on the main texts.

We identified 853 publications of which 103 met our criteria for inclusion (Fig 1 and S1 File). 79 publications (77%) used mice and 24 (23%) used rats. 67 publications (65%) used male animals, 9 (9%) used females, 17 (16%) used mixed populations, and gender was not reported in 10 (10%) publications. At the initiation of treatment 43 animals (42%) were juvenile, 26 (25%) were adult, 10 (10%) were mature and age was not reported in 24 (23%) publications.

74 articles (72%) used subcutaneous administration, with 27 (26%) using intraperitoneal, oral in 1 (1%), and was not stated in 1 study (1%). We categorised the dose of d-galactose into 4 groups: 0–60 mg/kg (17%). 100–125 mg/kg (35%), 150–250 mg/kg (27%) and 300–1250 mg/kg (20%). We categorized the duration of d-galactose exposure used to induce ageing as 7–40 days (10%), 42 days (22%), 49–56 days (45%) and 60–112 days (23%).

78 studies reported neurobehavioural outcomes, all related to cognition; 43 (55%) articles used Morris water maze (MWM) test to evaluate cognition-related neurobehavioral outcome, with 21 (26.%) using the shuttle box task, 7 (9%) Y-maze test, 5 (6%) novel object recognition (NOR) test, and one each for the T-maze test and the radial arm maze test. 60 studies reported neurochemical outcomes; 15 (25%) evaluated protein carbonyl, 15 (25%) measured AChE, 11 TNF-α, 7 IL-1, 5 IL-6, 4 Bax and 3 Bcl-2.

### Global estimates of impairment in the neurobehavioral and neurochemical scores

**Fig 2:**
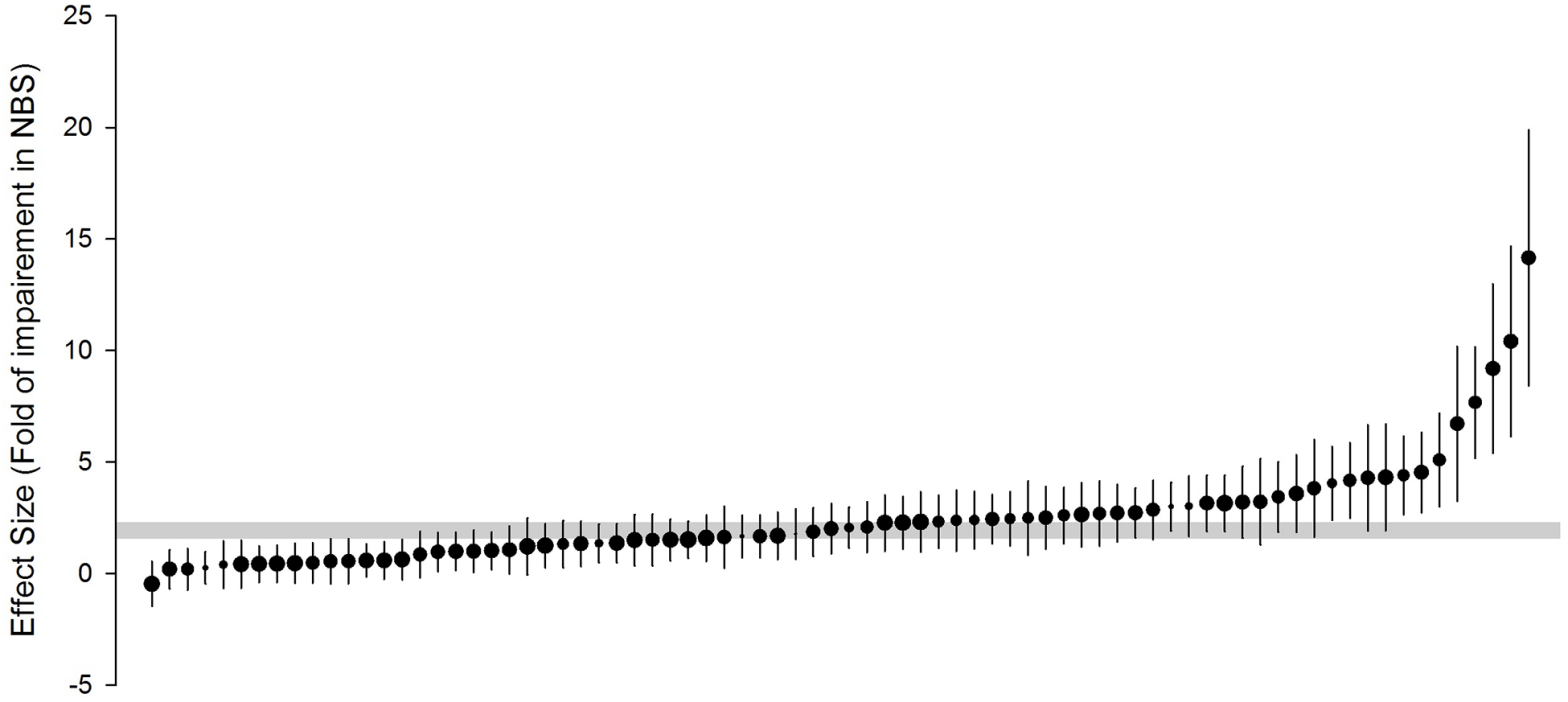
Timber plot of the effect size for each of the included comparisons evaluating impairment in the neurobehavioral score (NBS). Vertical error bars represent 95% confidence intervals (CI) and horizontal grey bars show the 95% CI of the global estimate of impairment in the NBS. The relative number of animals used in each comparison has been represented by symbol sizes.

Overall, d-galactose administration in rodents impaired cognition-related outcomes by 1.79 SMD (95% confidence interval (CI), 1.49 to 2.08, 78 comparisons; Fig 2). There was substantial heterogeneity between studies (χ^2^ = 382.37, *I*^2^ = 79.9%, degree of freedom (d.f.) = 77, *p* <0.0005). D-galactose administration also resulted in neurochemical changes consistent with ageing (effect size 3.20 SMD, 95% confidence interval (CI) 2.95 to 3.44, 250 comparisons, S2 File). Again, there was substantial heterogeneity between studies (χ^2^ = 1517.45, *I*^2^ = 83.6%, d.f. = 249, *p* <0.0005).

### Study characteristics

#### Neurobehavioral score

**Fig 3:**
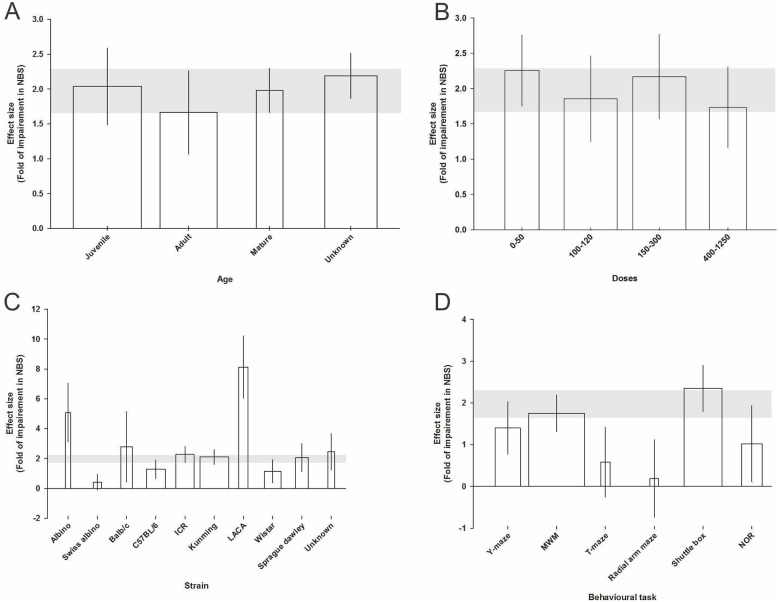
Significant effect of animal age category (A), d-galactose dose category (B), animal strain (C), and behavioural task (D) on the neurobehavioral score (NBS) measured using standardised mean differences (SMDs). Horizontal grey bars show the 95% CI of the global estimate of impairment in NBS and vertical error bars show 95% confidence interval (CI). The relative number of animals in each comparison has been presented using bar width.

After excluding experiments in which the age of the animal was not stated the mature group had the largest (2.14 SMD, 95% CI, 0.91 to 3.37) and the adult group had the smallest (1.66 SMD, 95% CI, 1.06 to 2.25: χ^2^ for difference = 304.26, Τ^2^ = 1.07, *I*^2^ = 81.9%, d.f. = 55, *p*<0.0005) change in the cognition-related NBS (Fig 3A).

There was no dose- response effect; the largest impairment was found with 0–50 mg/kg of d-galactose (2.25 SMD, 95% CI, -1.75 to 2.76) and the smallest with 400–1250 mg/kg (1.73 SMD, 95% CI, 1.15 to 2.31: χ^2^ for difference = 304.26, Τ^2^ = 1.07, *I*^2^ = 81.9%, d.f. = 55, *p*<0.0005; Fig 3B).

The largest effect was in LACA strains (8.12 SMD, 95% CI, 6.03 to 10.21) and the smallest in Swiss Albino strains (0.41 SMD, 95% CI, -0.10 to 0.92: χ^2^ for difference = 347.15, Τ^2^ = 1.26, *I*^2^ = 84.2%, d.f. = 55, *p*<0.0005; Fig 3C).

The largest effect was seen in experiments using the shuttle box task detected the highest (2.34 SMD, 95% CI, 1.7 to 2.89) and the smallest in those using the radial arm maze task (0.19 SMD, 95% CI, -0.73 to 1.11: χ^2^ for difference = 339.48, Τ^2^ = 1.12, *I*^2^ = 77.3%, d.f. = 77, *p*<0.0005; Fig 3D). We found no significant effect on the impairment in NBS of the model duration, route of administration, animal gender or rodent type (mouse versus rat).

#### Oxidative stress-related neurochemical changes

**Fig 4:**
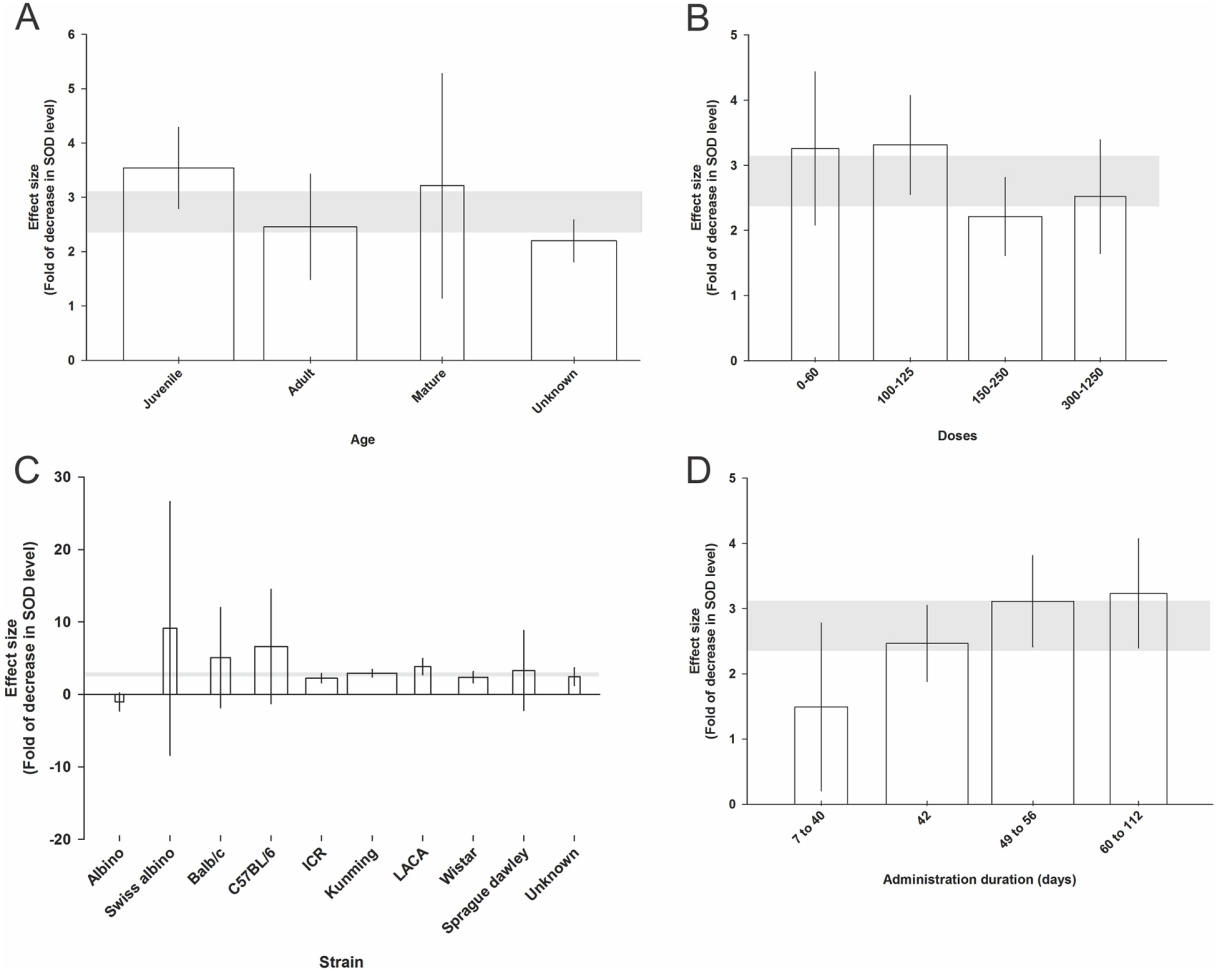
Significant effect of animal age category (A), d-galactose dose category (B), animal strain (C), and administration duration (D) on the decrease in superoxide dismutase (SOD) level measured using standardised mean differences (SMDs). Horizontal grey bars show the 95% CI of the global estimate of the decrease in SOD level and vertical error bars show 95% confidence interval (CI). The relative number of animals in each comparison has been presented using bar width.

The juvenile group showed the largest (3.54 SMD, 95% CI, 2.79 to 4.29) and the group where age was not stated the smallest (2.20 SMD, 95% CI, 1.81 to 2.58), reduction in the abundance of SOD (χ^2^ = 283.09, Τ^2^ = 1.73, *I*^2^ = 78.8%, d.f. = 60, *p*<0.0005; Fig 4A).

For dose, animals receiving 100-125mg/kg dose had the largest (3.31 SMD, 95% CI, 2.55 to 4.07) and those receiving 150–250 mg/kg the smallest (2.21 SMD, 95% CI, 1.61 to 2.81) reduction in the abundance of SOD (χ^2^ = 283.09, Τ^2^ = 1.73, *I*^2^ = 78.8%, d.f. = 60, *p*<0.0005; Fig 4B).

Swiss Albino strain showed the largest (9.10 SMD, 95% CI, -8.39 to 26.61) and Albino the smallest (1.04 SMD, 95% CI, -2.28 to 0.19, χ^2^ for difference = 283.09, Τ^2^ = 1.73, *I*^2^ = 78.8%, d.f. = 60, *p*<0.0005; Fig 4C) change in SOD level. The administration duration of 60–112 days had the highest (3.23 SMD, 95% CI, 2.39 to 4.07) and 7–40 days the lowest (1.49 SMD, 95% CI, 0.20 to 2.78, χ^2^ for difference = 283.09, Τ^2^ = 1.73, *I*^2^ = 78.8%, d.f. = 60, *p*<0.0005; Fig 4D) impact on the fold of decrease in SOD level.

We found no significant impact of the route of administration, animal gender and rodent type on the change in the SOD level.

**Fig 5:**
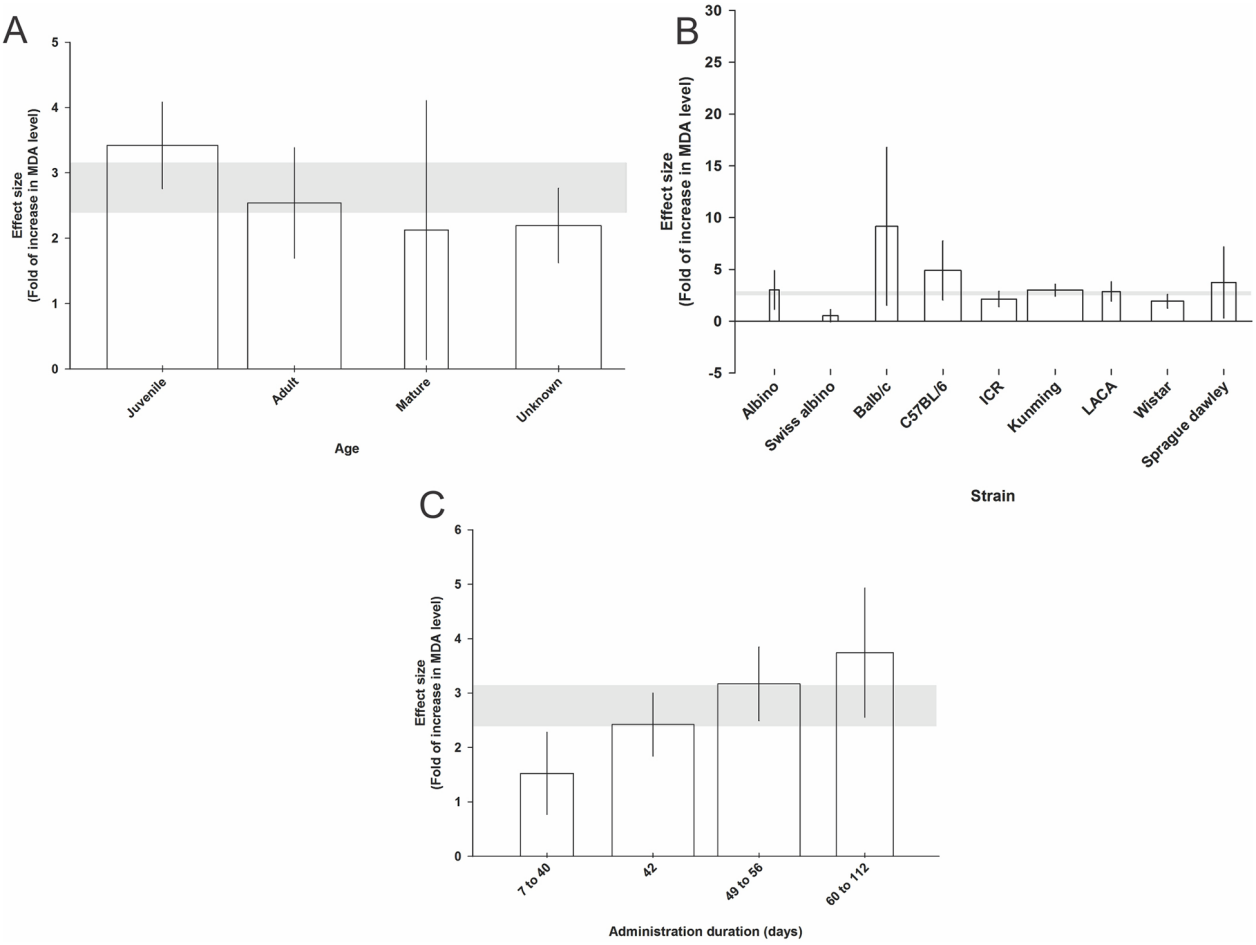
Significant effect of animal age category (A), animal strain (B), and administration duration (C) on the malondialdehyde (MDA) level measured using standardised mean differences (SMDs). Horizontal grey bars show the 95% CI of the global estimate of the increase in MDA level and vertical error bars show 95% confidence interval (CI). The relative number of animals in each comparison has been presented using bar width.

For MDA, the juvenile age category had the largest (3.42 SMD, 95% CI, 2.75 to 4.08) and mature animals the smallest (2.12 SMD, 95% CI, 0.14 to 4.10) change in MDA levels (χ^2^ for difference = 335.91, Τ^2^ = 1.90, *I*^2^ = 80.6%, d.f. = 65, *p*<0.0005; Fig 5A). The Balb/c strain showed the largest (9.15 SMD, 95% CI, 1.53 to 16.78) and Swiss Albino the smallest (0.53 SMD, 95% CI, 0.05 to 1.12) change in MDA level (χ^2^ = 335.91, Τ^2^ = 1.90, *I*^2^ = 80.6%, d.f. = 65, *p*<0.0005; Fig 5B). Duration of administration of 60–112 days had the largest (3.74 SMD, 95% CI, 2.55 to 4.93) and 7–40 days the smallest (1.52 SMD, 95% CI, 0.76 to 2.28) change in MDA level (χ^2^ = 283.09, Τ^2^ = 1.73, *I*^2^ = 78.8%, d.f. = 60, *p*<0.0005; Fig 5C). We found no significant impact of the route of administration, drug dose category, animal gender and rodent type on the change in MDA level.

**Fig 6:**
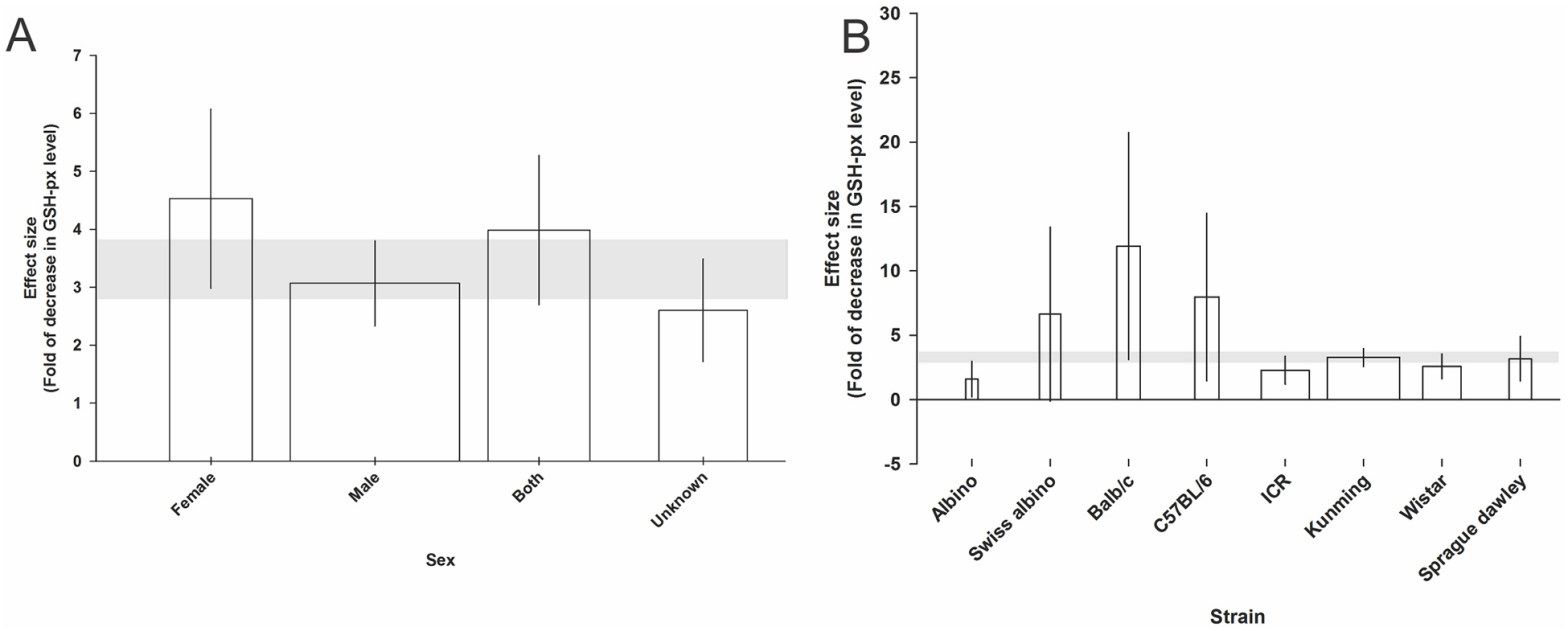
Significant effect of animal sex (A) and animal strain (B on the glutathione peroxidase (GSH-px) level measured using standardised mean differences (SMDs). Horizontal grey bars show the 95% CI of the global estimate of the decrease in GSH-px level and vertical error bars show 95% confidence interval (CI). The relative number of animals in each comparison has been presented using bar width.

For GSH-px, the effect was largest in females (4.52 SMD, 95% CI, 2.98 to 6.07) and the unknown group had the smallest (2.60 SMD, 95% CI, 1.71 to 3.48) effect on GSH-px levels (χ^2^ = 234.85, Τ^2^ = 2.39, *I*^2^ = 80.8%, d.f. = 45, *p*<0.0005; Fig 6A). The Balb/c strain showed the largest (11.92 SMD, 95% CI, 3.10 to 20.74) and Albino the smallest (1.58 SMD, 95% CI, 0.21 to 2.96) effect on GSH-px (χ^2^ = 234.85, Τ^2^ = 2.39, *I*^2^ = 80.8%, d.f. = 45, *p*<0.0005; Fig 6B). Stratifying the data according to the dose of drug, model duration, rodent type, route of administration and animal age, had no significant impact on the change in GSH-px level.

#### Other neurochemical scores

This systematic review and meta-analysis did not show a statistically significant impact of the study design indexes in d-galactose model of ageing on other studied neurochemical factors including PCs, AChE, Bcl-2, Bax, IL-1, IL-6, and TNF-α.

### Study quality

**Fig 7:**
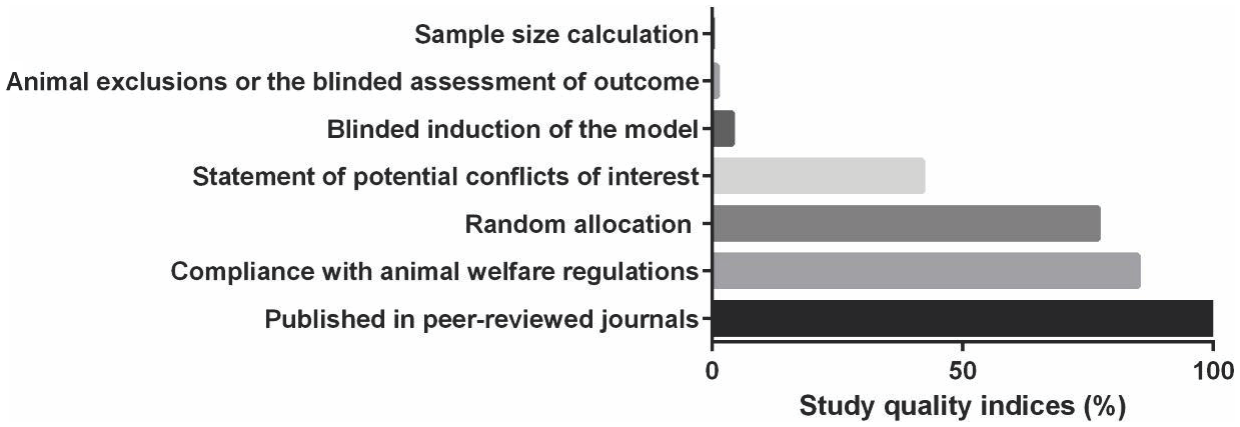
Percentage of included studies for each item in modified version of the CAMARADES' study quality checklist.

All of the articles had been published in peer-reviewed journals. Forty-three (42%) publications had statement of potential conflicts of interest. Eighty-eight (85%) articles reported compliance with animal welfare regulations. Random allocation to group was reported in 79 (77%) studies. Four (4%) studies reported blinded induction of the model. No study reported a sample size calculation method and only one study each (1%) study reported either animal exclusions or the blinded assessment of outcome (Fig 7). There was no statistically significant impact of these items on reported effect sizes for behavioural or neurochemical outcomes.

## Discussion

Study designs in the d-galactose model of ageing appear to have a significant but inconsistent impact on the cognition-related neurobehavioral scores and on neurochemical outcomes including SOD, MDA, and GSH-px. However, we did not see effects of factors such as age, gender, dose for other neurochemical outcomes such as PCs, Bax, Bcl-2, AChE, IL-1, 6 and TNF-α. This may be due to the low number of publications assessing these outcomes. Although these findings support the ability of d-galactose treatment to model features of brain ageing, our findings should be interpreted with some caution because of the limitations of the present study and of the included publications.

### Study quality and clinical relevance

We used a modified CAMARADES’ study quality checklist to evaluate the internal and external validity of the included publications. This checklist encompassed items such as random allocation to group (model/sham), blinded model induction, blinded assessment of outcome, sample size calculation, compliance with animal welfare regulations, statement of potential conflicts of interest, reporting of animal exclusions, and publication in peer reviewed journal. We and others have previously shown that publications with low methodological quality have a tendency to overstate effect sizes [19]. The quality of publications included in this meta-analysis was only modest (a median 3 out of 8 checklist items were present). Important meaures to reduce the risk of bias such as blinded induction of the model, blinded assessment of the outcome, sample size calculation and reporting of animal exclusions were all reported only rarely. A further concern is the remarkable heterogeneity between studies, which suggests the presence of other factors driving the effects seen. Identification of these factors would be important to better define the optimum use of this model, particularly if it to be used as the basis of selecting drugs for clinical trials. Future studies should also report measures to reduce the risk of bias, such as those included in the study quality checklist developed by CAMARADES for animal studies quality assessment or tfor example that proposed by Downs and Black [20].

### Study design

It has been shown that increased oxidative stress is strongly related to impaired memory and biomarkers of oxidative stress are associated with cognitive outcomes [21, 22], so recapitulating oxidative stress in animal models is imprortant.

Here we found that administration of d-galactose at a dose of 0–50 mg/kg had the largest effect on the impairment of NBS in the rodent. However, the effect on SOD—the only neurochemical outcome where an effect of dose was apparent–occurred at doses between 100–125 mg/kg.

We also found that d-galactose-induced impairment in the NBS was maximal in the mature rodent, but effects on SOD and MDA were highest in the juvenile group. This may be due to the small number of observations, or reflect a delay between the neurochemical and neurobehavioural effects of d-galactose.

Further, in our dataset, we saw that animal strain is an important factor in both d-galactose-induced NBS and neurochemical outcome impairment, where the LACA strain showed the highest impairment in NBS. Experiments using Swiss albino showed the largest effect on SOD, those using Balb/c the largest effects on GSH-px and MDA levels. Also, the different number of animals used in each group might affect the results as studies with a small number of animals giving imprecise results.

In addition, the highest decrease and increase in SOD and MDA levels respectively were in administration duration of 60–112 days, and this may reflect the prolonged period of d-galactose administration compared with that for other outcomes reported.

However, this study failed to find a statistically significant impact of study quality and design factors on d-galactose model of ageing in other neurochemical outcomes including PCs, Bax, Bcl-2, AChE, IL-1, 6 and TNF-α. This may be due to the small number of studies evaluating these factors in the included publications.

### Potential limitations

Our meta-analysis had some potential limitations and its outcomes should be interpreted with caution. First, this study was observational and based on the results of existing published data; therefore our findings can be considered as hypothesis generating only. Second, the quality of the included articles was in general low, and because these studies tend to overstate outcomes we may have overestimated the effect sizes. Thirdly, it is possible that this literature is confounded by publication bias [23]. However, the use of conventional approaches to assess for the likelihood of publication bias performs poorly in small studies with SMD estimates of effect size, and so we elected not to proceed with this here. Finally, the power of stratified meta-analysis to detect the impact of independent variables is limited, and we estimate (based on simulation studies) power of only 20% to detect an impact of a 1 SMD difference in observed effect [24].

## Conclusion

Brain ageing research using d-galactose model in rodent, which mimics age-related cognitive impairment and oxidative stress, has recently gained a remarkable attention. Our results represented an overview of different aspects of the rodent d-galactose model and the neurobehavioral and neurochemical outcomes reported to better understand the characteristics of the model. This meta-analysis indicates the inconsistency and heterogeneity of the included publications, perhaps due to modest reported study quality or to other factors which influence the performance of the model which have not been identified here. These shortcomings should be addresses before efficacy in d-galactose models can be used as a signal to proceed with human clinical trials.

## Supporting information

S1 File The comprehensive list of included studies in the systematic review and meta-analysis. (PDF)

S2 File The effect sizes of the included studies in the systematic review and meta-analysis. (PDF)
