# Correction: D-galactose-induced brain ageing model: A systematic review and meta-analysis on cognitive outcomes and oxidative stress indices

**DOI:** 10.1371/journal.pone.0190328

**Published:** 2017-12-21

**Authors:** Saeed Sadigh-Eteghad, Alireza Majdi, Sarah K. McCann, Javad Mahmoudi, Manouchehr S. Vafaee, Malcolm R. Macleod

There are errors in the author affiliations. The affiliations should appear as shown here:

Saeed Sadigh-Eteghad^1^, Alireza Majdi^1^, Sarah K. McCann^2^, Javad Mahmoudi^1^, Manouchehr S. Vafaee^1,3,4^, Malcolm R. Macleod^5^

**1** Neurosciences Research Center (NSRC), Tabriz University of Medical Sciences, Tabriz, Iran, **2** Centre for Clinical Brain Sciences, University of Edinburgh, Edinburgh, Scotland, United Kingdom, **3** Department of Nuclear Medicine, Odense University Hospital, Odense, Denmark, **4** Department of Psychiatry, Clinical Institute, University of Southern Denmark, Odense, Denmark, **5** Department of Clinical Neurosciences, The University of Edinburgh, Edinburgh, United Kingdom.
