# Supplementary material for: D-galactose-induced brain ageing model: A systematic review and meta-analysis on cognitive outcomes and oxidative stress indices
Source: PLoS One. 2017 Aug 30;12(8):e0184122. doi: 10.1371/journal.pone.0184122 (PMC5576729; doi:10.1371/journal.pone.0184122)
Supplement: S1 File — (PDF) [file pone.0184122.s001.pdf]

Supplementary data 1

List of included studies

1 Gong Y-S, Guo J, Hu K, Gao Y-Q, Xie B-J, Sun Z-D, Yang E-N, Hou F-L: Ameliorative effect of lotus seedpod proanthocyanidins on cognitive impairment and brain aging induced by d-galactose. *Experimental gerontology* 2016; 74:21-28.

2 Rehman SU, Shah SA, Ali T, Chung JI, Kim MO: Anthocyanins reversed d-galactose-induced oxidative stress and neuroinflammation mediated cognitive impairment in adult rats. *Molecular neurobiology* 2017; 54:255-271.

3 Lei H, Wang B, Li W-P, Yang Y, Zhou A-W, Chen M-Z: Anti-aging effect of astragalosides and its mechanism of action. *Acta Pharmacologica Sinica* 2003; 24:230-234.

4 Ji DB, Ye J, Li CL, Wang YH, Zhao J, Cai SQ: Antiaging effect of cordyceps sinensis extract. *Phytotherapy Research* 2009; 23:116-122.

5 Chao P-c, Yin M-c, Mong M-c: Anti-apoptotic and anti-glycative effects of asiatic acid in the brain of d-galactose treated mice. *Food & function* 2015; 6:542-548.

6 Tsai S-j, Yin M-c: Anti-glycative and anti-inflammatory effects of protocatechuic acid in brain of mice treated by d-galactose. *Food and chemical toxicology* 2012; 50:3198-3205.

7 Qingming Y, Xianhui P, Weibao K, Hong Y, Yidan S, Li Z, Yanan Z, Yuling Y, Lan D, Guoan L: Antioxidant activities of malt extract from barley (hordeum vulgare l.) toward various oxidative stress in vitro and in vivo. *Food Chemistry* 2010; 118:84-89.

8 Zhong W, Liu N, Xie Y, Zhao Y, Song X, Zhong W: Antioxidant and anti-aging activities of mycelial polysaccharides from lepista sordida. *International journal of biological macromolecules* 2013; 60:355-359.

9 An F, Yang G, Tian J, Wang S: Antioxidant effects of the orientin and vitexin in trollius chinensis bunge in d-galactose-aged mice. *Neural regeneration research* 2012; 7:2565.

10 Tsai S-j, Yin M-c: Anti-oxidative, anti-glycative and anti-apoptotic effects of oleanolic acid in brain of mice treated by d-galactose. *European journal of pharmacology* 2012; 689:81-88.

11 Han C-H, Lin Y-S, Lee T-L, Liang H-J, Hou W-C: Asn-trp dipeptides improve the oxidative stress and learning dysfunctions in d-galactose-induced balb/c mice. *Food & function* 2014; 5:2228-2236.

12 Wu W, Wang X, Xiang Q, Meng X, Peng Y, Du N, Liu Z, Sun Q, Wang C, Liu X: Astaxanthin alleviates brain aging in rats by attenuating oxidative stress and increasing bdnf levels. *Food & function* 2014; 5:158-166.

13 Wu W, Li M, Liu L, Gao J, Kong H, Ding J, Hu G, Xiao M: Astrocyte activation but not neuronal impairment occurs in the hippocampus of mice after 2weeks of d-galactose exposure. *Life sciences* 2011; 89:355-363.

14 Wei H, Li L, Song Q, Ai H, Chu J, Li W: Behavioural study of the d-galactose induced aging model in c57bl/6j mice. *Behavioural brain research* 2005; 157:245-251.

15 Wang C-Q, Yang G-Q: Betacyanins from portulaca oleracea l. Ameliorate cognition deficits and attenuate oxidative damage induced by d-galactose in the brains of senescent mice. *Phytomedicine* 2010; 17:527-532.

16 Çoban J, Doğan-Ekici I, Aydın AF, Betül-Kalaz E, Doğru-Abbasoğlu S, Uysal M: Blueberry treatment decreased d-galactose-induced oxidative stress and brain damage in rats. *Metabolic brain disease* 2015; 30:793-802.

17 Ullah F, Ali T, Ullah N, Kim MO: Caffeine prevents d-galactose-induced cognitive deficits, oxidative stress, neuroinflammation and neurodegeneration in the adult rat brain. *Neurochemistry international* 2015; 90:114-124.
18 Wei S, Shi W, Li M, Gao Q: Calorie restriction down-regulates expression of the iron regulatory hormone hepcidin in normal and d-galactose–induced aging mouse brain. *Rejuvenation research* 2014; 17:19-26.
19 Aydın AF, Çoban J, Doğan-Ekici I, Betül-Kalaz E, Doğru-Abbasoğlu S, Uysal M: Carnosine and taurine treatments diminished brain oxidative stress and apoptosis in d-galactose aging model. *Metabolic brain disease* 2016; 31:337-345.
20 Zhang XL, Jiang B, Li ZB, Hao S, An LJ: Catalpol ameliorates cognition deficits and attenuates oxidative damage in the brain of senescent mice induced by d-galactose. *Pharmacology Biochemistry and Behavior* 2007; 88:64-72.
21 Zhang X, Jin C, Li Y, Guan S, Han F, Zhang S: Catalpol improves cholinergic function and reduces inflammatory cytokines in the senescent mice induced by d-galactose. *Food and chemical toxicology* 2013; 58:50-55.
22 Lu J, Wu D-m, Hu B, Cheng W, Zheng Y-l, Zhang Z-f, Ye Q, Fan S-h, Shan Q, Wang Y-j: Chronic administration of troxerutin protects mouse brain against d-galactose-induced impairment of cholinergic system. *Neurobiology of learning and memory* 2010; 93:157-164.
23 Cui X, Zuo P, Zhang Q, Li X, Hu Y, Long J, Packer L, Liu J: Chronic systemic d-galactose exposure induces memory loss, neurodegeneration, and oxidative damage in mice: Protective effects of r-α-lipoic acid. *Journal of neuroscience research* 2006; 83:1584-1590.
24 Tikhonova MA, Yu C-H, Kolosova NG, Gerlinskaya LA, Maslennikova SO, Yudina AV, Amstislavskaya TG, Ho Y-J: Comparison of behavioral and biochemical deficits in rats with hereditary defined or d-galactose-induced accelerated senescence: Evaluating the protective effects of diosgenin. *Pharmacology Biochemistry and Behavior* 2014; 120:7-16.
25 Li Z, Zhang Z, Zhang J, Jia J, Ding J, Luo R, Liu Z: Cordyceps militaris extract attenuates d-galactose-induced memory impairment in mice. *Journal of medicinal food* 2012; 15:1057-1063.
26 Banji OJ, Banji D, Ch K: Curcumin and hesperidin improve cognition by suppressing mitochondrial dysfunction and apoptosis induced by d-galactose in rat brain. *Food and Chemical Toxicology* 2014; 74:51-59.
27 Banji D, Banji OJ, Dasaroju S, CH KK: Curcumin and piperine abrogate lipid and protein oxidation induced by d-galactose in rat brain. *Brain research* 2013; 1515:1-11.
28 Lan Z, Liu J, Chen L, Fu Q, Luo J, Qu R, Kong L, Ma S: Danggui-shaoyao-san ameliorates cognition deficits and attenuates oxidative stress-related neuronal apoptosis in d-galactose-induced senescent mice. *Journal of ethnopharmacology* 2012; 141:386-395.
29 Zhang X-l, An L-j, Bao Y-m, Wang J-y, Jiang B: D-galactose administration induces memory loss and energy metabolism disturbance in mice: Protective effects of catalpol. *Food and chemical toxicology* 2008; 46:2888-2894.
30 Cardoso A, Magano S, Marrana F, Andrade JP: D-galactose high-dose administration failed to induce accelerated aging changes in neurogenesis, anxiety, and spatial memory on young male wistar rats. *Rejuvenation research* 2015; 18:497-507.
31 Chiu C-S, Chiu Y-J, Wu L-Y, Lu T-C, Huang T-H, Hsieh M-T, Lu C-Y, Peng W-H: Diosgenin ameliorates cognition deficit and attenuates oxidative damage in senescent mice induced by d-galactose. *The American journal of Chinese medicine* 2011; 39:551-563.
32 Turgut NH, Mert DG, Kara H, Egilmez HR, Arslanbas E, Tepe B, Gungor H, Yilmaz N, Tuncel NB: Effect of black mulberry (morus nigra) extract treatment on cognitive impairment and oxidative stress status of d-galactose-induced aging mice. *Pharmaceutical biology* 2016; 54:1052-1064.

33 Piao L, Zhang X, Jin X, Li S: Effect of boschniakia rossica extract on free radicals in brain of d-galactose induced senile rats. *Zhong xi yi jie he xue bao= Journal of Chinese integrative medicine* 2003; 1:125-127.
34 Wang D, Liu M, Cao J, Cheng Y, Zhuo C, Xu H, Tian S, Zhang Y, Zhang J, Wang F: Effect of colla corii asini (e'jiao) on d-galactose induced aging mice. *Biological and Pharmaceutical Bulletin* 2012; 35:2128-2132.
35 Yi Z-J, Fu Y-R, Li M, Gao K-S, Zhang X-G: Effect of lta isolated from bifidobacteria on d-galactose-induced aging. *Experimental gerontology* 2009; 44:760-765.
36 Xu X, Zhang Z: Effect of puerarin on learning-memory behavior and synaptic structure of hippocampus in the aging mice induced by d-galactose. *Yao xue xue bao= Acta pharmaceutica Sinica* 2002; 37:1-4.
37 Liu J, Yu H, Ning X: Effect of quercetin on chronic enhancement of spatial learning and memory of mice. *Science in China Series C: Life Sciences* 2006; 49:583-590.
38 Zhan G, Yang N, Xiao B: The effect of wu-he dipsacus asper on mice-aging model induced by d-galactose. *Zhongguo ying yong sheng li xue za zhi= Zhongguo yingyong shenglixue zazhi= Chinese journal of applied physiology* 2014; 30:174-177.
39 Hailiqian T, Kang J, Sun L: Effects of aqueous extract of hedysarum austrosibiricum on metabolism of oxyen free radicals in subacute aging mice caused by d-galactose. *Zhongguo Zhong yao za zhi= Zhongguo zhongyao zazhi= China journal of Chinese materia medica* 2007; 32:729-731.
40 Chen W, Yang Q, Wei X: Effects of chrysalis oil on learning, memory and oxidative stress in d-galactose-induced ageing model of mice. *Zhejiang da xue xue bao Yi xue ban= Journal of Zhejiang University Medical sciences* 2013; 42:620-624.
41 Nam SM, Choi JH, Yoo DY, Kim W, Jung HY, Kim JW, Yoo M, Lee S, Kim CJ, Yoon YS: Effects of curcumin (curcuma longa) on learning and spatial memory as well as cell proliferation and neuroblast differentiation in adult and aged mice by upregulating brain-derived neurotrophic factor and creb signaling. *Journal of medicinal food* 2014; 17:641-649.
42 Ren S, He X, Yun S, Zhang S, Xiao Z, Wei S: Effects of exercise on spatial learning and hippocampal synaptic plasticity in brain aging mice. *Wei sheng yan jiu= Journal of hygiene research* 2010; 39:239-241.
43 Xu X-H: Effects of puerarin on fatty superoxide in aged mice induced by d-galactose. *Zhongguo Zhong yao za zhi= Zhongguo zhongyao zazhi= China journal of Chinese materia medica* 2003; 28:66-69.
44 Cui Y, Zhang B, Zhang R, Li C, Zhao Y, Ren Y, Yang J: Effects of resveratrol on morphology and oxidative stress of brain tissues in aging mice. *Wei sheng yan jiu= Journal of hygiene research* 2013; 42:995-998, 1003.
45 Han C-H, Lin Y-F, Lin Y-S, Lee T-L, Huang W-J, Lin S-Y, Hou W-C: Effects of yam tuber protein, dioscorin, on attenuating oxidative status and learning dysfunction in d-galactose-induced balb/c mice. *Food and Chemical Toxicology* 2014; 65:356-363.
46 Yu Y, Bai F, Wang W, Liu Y, Yuan Q, Qu S, Zhang T, Tian G, Li S, Li D: Fibroblast growth factor 21 protects mouse brain against d-galactose induced aging via suppression of oxidative stress response and advanced glycation end products formation. *Pharmacology biochemistry and behavior* 2015; 133:122-131.
47 Chae C-H, Kim H-T: Forced, moderate-intensity treadmill exercise suppresses apoptosis by increasing the level of ngf and stimulating phosphatidylinositol 3-kinase signaling in the hippocampus of induced aging rats. *Neurochemistry international* 2009; 55:208-213.
48 Hsia C-H, Wang C-H, Kuo Y-W, Ho Y-J, Chen H-L: Fructo-oligosaccharide systemically diminished d-galactose-induced oxidative molecule damages in balb/cj mice. *British Journal of Nutrition* 2012; 107:1787-1792.
49 Li W-J, Nie S-P, Xie M-Y, Yu Q, Chen Y, He M: Ganoderma atrum polysaccharide attenuates oxidative stress induced by d-galactose in mouse brain. *Life sciences* 2011; 88:713-718.

50 Li W-J, Nie S-P, Peng X-P, Liu X-Z, Li C, Chen Y, Li J-E, Song W-R, Xie M-Y: Ganoderma atrum polysaccharide improves age-related oxidative stress and immune impairment in mice. *Journal of agricultural and food chemistry* 2012; 60:1413-1418.
51 Haider S, Liaquat L, Shahzad S, Sadir S, Madiha S, Batool Z, Tabassum S, Saleem S, Naqvi F, Perveen T: A high dose of short term exogenous d-galactose administration in young male rats produces symptoms simulating the natural aging process. *Life sciences* 2015; 124:110-119.
52 Tian Y, Zou B, Yang L, Xu S-f, Yang J, Yao P, Li C-m: High molecular weight persimmon tannin ameliorates cognition deficits and attenuates oxidative damage in senescent mice induced by d-galactose. *Food and chemical toxicology* 2011; 49:1728-1736.
53 Lei M, Hua X, Xiao M, Ding J, Han Q, Hu G: Impairments of astrocytes are involved in the d-galactose-induced brain aging. *Biochemical and biophysical research communications* 2008; 369:1082-1087.
54 Jin S-l, Yin Y-g: In vivo antioxidant activity of total flavonoids from indocalamus leaves in aging mice caused by d-galactose. *Food and chemical toxicology* 2012; 50:3814-3818.
55 Ye Y, Jia R-r, Tang L, Chen F: In vivo antioxidant and anti-skin-aging activities of ethyl acetate extraction from idesia polycarpa defatted fruit residue in aging mice induced by d-galactose. *Evidence-Based Complementary and Alternative Medicine* 2014; 2014
56 Lu Y, Ye M, Song S, Li L, Shaikh F, Li J: Isolation, purification, and anti-aging activity of melanin from lachnum singerianum. *Applied biochemistry and biotechnology* 2014; 174:762-771.
57 Xian Y-F, Su Z-R, Chen J-N, Lai X-P, Mao Q-Q, Cheng CH, Ip S-P, Lin Z-X: Isorhynchophylline improves learning and memory impairments induced by d-galactose in mice. *Neurochemistry international* 2014; 76:42-49.
58 Liu Y, Zhang Z, Yang X: Kinetin protects against lipid peroxidation and improves antioxidant status in cultured astrocytes and mouse brain exposed to d-galactose. *African Journal of Biotechnology* 2011; 10:11721.
59 Woo J-Y, Gu W, Kim K-A, Jang S-E, Han MJ, Kim D-H: Lactobacillus pentosus var. Plantarum c29 ameliorates memory impairment and inflammaging in a d-galactose-induced accelerated aging mouse model. *Anaerobe* 2014; 27:22-26.
60 Yan T, Shang L, Wang M, Zhang C, Zhao X, Bi K, Jia Y: Lignans from schisandra chinensis ameliorate cognition deficits and attenuate brain oxidative damage induced by d-galactose in rats. *Metabolic brain disease* 2016; 31:653-661.
61 Li J-J, Zhu Q, Lu Y-P, Zhao P, Feng Z-B, Qian Z-M, Zhu L: Ligustilide prevents cognitive impairment and attenuates neurotoxicity in d-galactose induced aging mice brain. *Brain research* 2015; 1595:19-28.
62 Ali T, Badshah H, Kim TH, Kim MO: Melatonin attenuates d-galactose-induced memory impairment, neuroinflammation and neurodegeneration via rage/nf-kb/jnk signaling pathway in aging mouse model. *Journal of pineal research* 2015; 58:71-85.
63 Yoo DY, Kim W, Lee CH, Shin BN, Nam SM, Choi JH, Won MH, Yoon YS, Hwang IK: Melatonin improves d-galactose-induced aging effects on behavior, neurogenesis, and lipid peroxidation in the mouse dentate gyrus via increasing pcreb expression. *Journal of pineal research* 2012; 52:21-28.
64 Shen YX, Xu SY, Wei W, Sun XX, Yang J, Liu LH, Dong C: Melatonin reduces memory changes and neural oxidative damage in mice treated with d-galactose. *Journal of pineal research* 2002; 32:173-178.
65 Fu C, Wang T, Wang Y, Chen X, Jiao J, Ma F, Zhong M, Bi K: Metabonomics study of the protective effects of green tea polyphenols on aging rats induced by d-galactose. *Journal of pharmaceutical and biomedical analysis* 2011; 55:1067-1074.
66 Ma W, Yuan L, Yu H, Xi Y, Xiao R: Mitochondrial dysfunction and oxidative damage in the brain of diet-induced obese rats but not in diet-resistant rats. *Life sciences* 2014; 110:53-60.

67 Zhou Y, Dong Y, Xu Q, He Y, Tian S, Zhu S, Zhu Y, Dong X: Mussel oligopeptides ameliorate cognition deficit and attenuate brain senescence in d-galactose-induced aging mice. *Food and chemical toxicology* 2013; 59:412-420.

68 Fu W, Du G, Liu D, Ruan J-L: Neuroprotective effect of a caffeic acid derivative from abacopteris penangiana. *Pharmaceutical biology* 2013; 51:376-382.

69 Ji Z-H, Liu C, Zhao H, Yu X-Y: Neuroprotective effect of biatractylenolide against memory impairment in d-galactose-induced aging mice. *Journal of Molecular Neuroscience* 2015; 55:678-683.

70 Ambikar DB, Harle UN, Khandare RA, Bore VV, Vyawahare N: Neuroprotective effect of hydroalcoholic extract of dried fruits of trapa bispinosa roxb on lipofuscinogenesis and fluorescence product in brain of d-galactose induced ageing accelerated mice. *Indian Journal of Experimental Biology* 2010:378-382.

71 He M, Zhao L, Wei M-J, Yao W-F, Zhao H-S, Chen F-J: Neuroprotective effects of (−)-epigallocatechin-3-gallate on aging mice induced by d-galactose. *Biological and Pharmaceutical Bulletin* 2009; 32:55-60.

72 Ruan Q, Hu X, Ao H, Ma H, Gao Z, Liu F, Kong D, Bao Z, Yu Z: The neurovascular protective effects of huperzine a on d-galactose-induced inflammatory damage in the rat hippocampus. *Gerontology* 2014; 60:424-439.

73 Lu J, Wu Dm, Hu B, Zheng Yl, Zhang Zf, Wang Yj: Ngf-dependent activation of trka pathway: A mechanism for the neuroprotective effect of troxerutin in d-galactose-treated mice. *Brain Pathology* 2010; 20:952-965.

74 Budni J, Pacheco R, da Silva S, Garcez ML, Mina F, Bellettini-Santos T, de Medeiros J, Voss BC, Steckert AV, da Silva Valvassori S: Oral administration of d-galactose induces cognitive impairments and oxidative damage in rats. *Behavioural brain research* 2016; 302:35-43.

75 Zhong S-Z, Ge Q-H, Qu R, Li Q, Ma S-P: Paeonol attenuates neurotoxicity and ameliorates cognitive impairment induced by d-galactose in icr mice. *Journal of the Neurological Sciences* 2009; 277:58-64.

76 Fu W, Lei Y-f, Chen J-l, Xiong C-m, Zhou D-n, Wu G-h, Chen J, Cai Y-l, Ruan J-l: Parathelypteriside attenuates cognition deficits in d-galactose treated mice by increasing antioxidant capacity and improving long-term potentiation. *Neurobiology of learning and memory* 2010; 94:414-421.

77 Prakash A, Kumar A: Pioglitazone alleviates the mitochondrial apoptotic pathway and mito-oxidative damage in the d-galactose-induced mouse model. *Clinical and Experimental Pharmacology and Physiology* 2013; 40:644-651.

78 Banji D, Banji OJ, Dasaroju S, Annamalai A: Piperine and curcumin exhibit synergism in attenuating d-galactose induced senescence in rats. *European journal of pharmacology* 2013; 703:91-99.

79 Kumar A, Prakash A, Dogra S: Protective effect of curcumin (curcuma longa) against d-galactose-induced senescence in mice. *Journal of Asian natural products research* 2011; 13:42-55.

80 Lin X, Zhang S, Huang R, Wei L, Tan S, Liang C, Lv S, Chen Y, Liang S, Tian Y: Protective effect of madecassoside against cognitive impairment induced by d-galactose in mice. *Pharmacology Biochemistry and Behavior* 2014; 124:434-442.

81 Liu A, Ma Y, Zhu Z: Protective effect of selenoarginine against oxidative stress in d-galactose-induced aging mice. *Bioscience, biotechnology, and biochemistry* 2009; 73:1461-1464.

82 Qu Z, Zhang J, Yang H, Huo L, Gao J, Chen H, Gao W: Protective effect of tetrahydropalmatine against d-galactose induced memory impairment in rat. *Physiology & behavior* 2016; 154:114-125.

83 Anand KV, Jaabir M, Sultan M, Thomas PA, Geraldine P: Protective role of chrysin against oxidative stress in d-galactose-induced aging in an experimental rat model. *Geriatrics & gerontology international* 2012; 12:741-750.

84 Lu J, Wu Dm, Zheng Yl, Hu B, Zhang Zf: Purple sweet potato color alleviates d-galactose-induced brain aging in old mice by promoting survival of neurons via pi3k pathway and inhibiting cytochrome c-mediated apoptosis. *Brain Pathology* 2010; 20:598-612.

85 Shan Q, Lu J, Zheng Y, Li J, Zhou Z, Hu B, Zhang Z, Fan S, Mao Z, Wang Y-j: Purple sweet potato color ameliorates cognition deficits and attenuates oxidative damage and inflammation in aging mouse brain induced by d-galactose. *BioMed Research International* 2009; 2009

86 Wu D-m, Lu J, Zheng Y-l, Zhou Z, Shan Q, Ma D-f: Purple sweet potato color repairs d-galactose-induced spatial learning and memory impairment by regulating the expression of synaptic proteins. *Neurobiology of learning and memory* 2008; 90:19-27.

87 Sun SW, Yu HQ, Zhang H, Zheng YL, Wang JJ, Luo L: Quercetin attenuates spontaneous behavior and spatial memory impairment in d-galactose–treated mice by increasing brain antioxidant capacity. *Nutrition Research* 2007; 27:169-175.

88 Lu J, Zheng Y-l, Luo L, Wu D-m, Sun D-x, Feng Y-j: Quercetin reverses d-galactose induced neurotoxicity in mouse brain. *Behavioural brain research* 2006; 171:251-260.

89 Xuan G, Liu C: Research on the effect of phenylethanoid glycosides (peg) of the cistanche deserticola on anti-aging in aged mice induced by d-galactose. *Zhong yao cai= Zhongyaocai= Journal of Chinese medicinal materials* 2008; 31:1385-1388.

90 Yang Y-C, Lin H-Y, Su K-Y, Chen C-H, Yu Y-L, Lin C-C, Yu S-L, Yan H-Y, Su K-J, Chen Y-LS: Rutin, a flavonoid that is a main component of saussurea involucrata, attenuates the senescence effect in d-galactose aging mouse model. *Evidence-Based Complementary and Alternative Medicine* 2012; 2012

91 Wang T, Di G, Yang L, Dun Y, Sun Z, Wan J, Peng B, Liu C, Xiong G, Zhang C: Saponins from panax japonicus attenuate d-galactose-induced cognitive impairment through its anti-oxidative and anti-apoptotic effects in rats. *Journal of Pharmacy and Pharmacology* 2015; 67:1284-1296.

92 Zhu SY, Dong Y, Tu J, Zhou Y, Zhou XH, Xu B: Silybum marianum oil attenuates oxidative stress and ameliorates mitochondrial dysfunction in mice treated with d-galactose. *Pharmacognosy magazine* 2014; 10:92.

93 Hsieh H-M, Wu W-M, Hu M-L: Soy isoflavones attenuate oxidative stress and improve parameters related to aging and alzheimer's disease in c57bl/6j mice treated with d-galactose. *Food and Chemical Toxicology* 2009; 47:625-632.

94 Prisila Dulcy C, Singh HK, Preethi J, Emmanuvel Rajan K: Standardized extract of bacopa monniera (beseb cdri-08) attenuates contextual associative learning deficits in the aging rat's brain induced by d-galactose. *Journal of neuroscience research* 2012; 90:2053-2064.

95 Wei H, Cai Y, Chu J, Li C, Li L: Temporal gene expression profile in hippocampus of mice treated with d-galactose. *Cellular and molecular neurobiology* 2008; 28:781-794.

96 Chen C, Lang S, Zuo P, Yang N, Wang X: Treatment with dehydroepiandrosterone increases peripheral benzodiazepine receptors of mitochondria from cerebral cortex in d-galactose-induced aged rats. *Basic & clinical pharmacology & toxicology* 2008; 103:493-501.

97 Lu J, Zheng Y-L, Wu D-M, Luo L, Sun D-X, Shan Q: Ursolic acid ameliorates cognition deficits and attenuates oxidative damage in the brain of senescent mice induced by d-galactose. *Biochemical pharmacology* 2007; 74:1078-1090.

98 Lu J, Wu D-m, Zheng Y-l, Hu B, Zhang Z-f, Ye Q, Liu C-m, Shan Q, Wang Y-j: Ursolic acid attenuates d-galactose-induced inflammatory response in mouse prefrontal cortex through inhibiting ages/rage/nf-κb pathway activation. *Cerebral Cortex* 2010:bhq002.

99 Nam SM, Choi JH, Yoo DY, Kim W, Jung HY, Kim JW, Kang S-Y, Park J, Kim D-W, Kim WJ: Valeriana officinalis extract and its main component, valerenic acid, ameliorate d-galactose-induced reductions in memory, cell proliferation, and neuroblast differentiation by reducing corticosterone levels and lipid peroxidation. *Experimental gerontology* 2013; 48:1369-1377.

100 Chiu C-S, Deng J-S, Hsieh M-T, Fan M-J, Lee M-M, Chueh F-S, Han C-K, Lin Y-C, Peng W-H: Yam (dioscorea pseudojaponica yamamoto) ameliorates cognition deficit and attenuates oxidative damage in senescent mice induced by d-galactose. *The American journal of Chinese medicine* 2009; 37:889-902.

101 MinháDoan V, PhucáNguyen V: Yulangsan polysaccharide improves redox homeostasis and immune impairment in d-galactose-induced mimetic aging. *Food & function* 2015; 6:1712-1718.
102 Wang W, Li S, Dong H-p, Lv S, Tang Y-y: Differential impairment of spatial and nonspatial cognition in a mouse model of brain aging. *Life sciences* 2009; 85:127-135.
103 Shinde V, Dhalwal K, Paradkar A, Mahadik K: Effects of human placental extract on age related antioxidant enzyme status in d-galactose treated mice. *Pharmacologyonline* 2007; 1:252-261.
