# Supplementary material for: D-galactose-induced brain ageing model: A systematic review and meta-analysis on cognitive outcomes and oxidative stress indices
Source: PLoS One. 2017 Aug 30;12(8):e0184122. doi: 10.1371/journal.pone.0184122 (PMC5576729; doi:10.1371/journal.pone.0184122)
Supplement: S2 File — (PDF) [file pone.0184122.s002.pdf]

Study	ES	[95% Conf. Interval]		% Weight
1	-2.303	-1.119	-3.488	0.5
2	4.747	7.325	2.168	0.35
3	1.144	2.105	0.182	0.52
4	5.199	7.493	2.904	0.38
5	4.343	6.334	2.352	0.41
6	4.231	5.936	2.525	0.44
7	1.221	2.315	0.126	0.51
8	3.338	4.991	1.684	0.45
9	1.258	2.236	0.279	0.52
10	8.323	11.332	5.314	0.3
11	2.814	4.123	1.504	0.49
12	5.853	7.833	3.873	0.41
13	1.458	2.276	0.64	0.53
14	2.705	3.863	1.547	0.5
15	5.861	11.742	-0.02	0.13
16	2.538	3.947	1.129	0.48
17	15.29	20.65	9.93	0.15
18	2.687	4.14	1.234	0.47
19	3.036	6.376	-0.304	0.27
20	42.327	56.991	27.663	0.03
21	1.961	4.441	-0.518	0.36
22	1.46	2.471	0.448	0.51
23	20.053	27.044	13.063	0.1
24	0.421	1.683	-0.841	0.49
25	2.239	3.566	0.913	0.48
26	-7.399	-4.512	-10.285	0.32
27	4.296	6.68	1.913	0.37
28	3.238	6.751	-0.274	0.26
29	1.385	2.194	0.577	0.53
30	1.271	2.066	0.477	0.53
31	5.089	8.853	1.324	0.24
32	7.357	10.048	4.666	0.34
33	2.326	3.929	0.723	0.45
34	6.734	9.594	3.873	0.32
35	11.648	23.009	0.287	0.04
36	6.97	9.535	4.405	0.35
37	2.948	6.214	-0.318	0.28
38	58.503	91.462	25.543	0.01
39	8.965	12.674	5.255	0.25
40	2.498	3.61	1.385	0.51
41	1.242	2.246	0.238	0.52
42	1.152	2.028	0.276	0.53
43	2.284	3.622	0.946	0.48
44	1.589	2.961	0.217	0.48
45	5.175	7.168	3.182	0.41
46	31.965	43.054	20.876	0.04
47	2.889	4.218	1.56	0.48
48	13.057	17.657	8.457	0.19

49	4.449	6.22	2.678	0.44
50	11.551	15.641	7.462	0.22
51	4.259	6.958	1.56	0.33
52	0.379	1.371	-0.612	0.52
53	1.688	2.741	0.635	0.51
54	2.341	3.695	0.987	0.48
55	2.26	3.592	0.928	0.48
56	3.55	5.62	1.48	0.4
57	4.884	6.787	2.981	0.42
58	4.117	6.424	1.811	0.38
59	2.354	3.551	1.158	0.5
60	8.387	11.874	4.899	0.26
61	4.526	6.32	2.732	0.43
62	10.364	14.615	6.113	0.21
63	4.814	6.971	2.658	0.39
64	4.642	6.591	2.694	0.42
65	6.238	8.722	3.754	0.36
66	2.461	4.649	0.273	0.39
67	3.021	4.385	1.657	0.48
68	2.025	3.147	0.903	0.5
69	2.811	4.054	1.569	0.49
70	6.764	9.901	3.628	0.29
71	4.909	7.559	2.259	0.34
72	1.735	3.148	0.321	0.47
73	3.446	5.473	1.418	0.41
74	1.818	3.038	0.599	0.49
75	1.819	3.257	0.38	0.47
76	2.28	3.227	1.334	0.52
77	4.415	5.811	3.019	0.48
78	0.641	1.815	-0.533	0.5
79	4.515	7.343	1.687	0.32
80	2.475	3.7	1.25	0.49
81	2.315	3.502	1.128	0.5
82	1.612	2.651	0.573	0.51
83	0.986	2.042	-0.07	0.51
84	9.195	13.824	4.566	0.19
85	5.917	8.143	3.691	0.38
86	8.577	11.67	5.484	0.3
87	3.142	4.734	1.551	0.46
88	3.04	4.409	1.671	0.48
89	1.373	2.834	-0.088	0.47
90	4.943	6.464	3.422	0.46
91	89.887	140.508	39.267	0
92	11.113	21.963	0.264	0.05
93	9.079	12.339	5.819	0.28
94	10.589	14.354	6.823	0.24
95	3.097	4.674	1.519	0.46
96	79.196	123.799	34.593	0
97	15.085	29.739	0.431	0.03
98	1.605	2.643	0.568	0.51

99	2.797	3.976	1.618	0.5
100	0.575	1.498	-0.349	0.52
101	1.026	1.887	0.165	0.53
102	3.813	5.623	2.003	0.43
103	3.017	4.874	1.16	0.43
104	2.415	3.626	1.205	0.5
105	7.452	10.584	4.321	0.29
106	1.728	2.69	0.765	0.52
107	7.175	9.806	4.543	0.34
108	12.204	16.514	7.893	0.2
109	2.467	3.691	1.244	0.49
110	16.473	22.238	10.709	0.14
111	3.832	6.32	1.344	0.36
112	0.212	1.195	-0.772	0.52
113	3.907	5.518	2.296	0.45
114	1.671	2.856	0.485	0.5
115	1.632	2.808	0.455	0.5
116	5.084	7.812	2.356	0.33
117	14.478	19.561	9.395	0.16
118	1.942	3.418	0.466	0.47
119	1.367	2.489	0.245	0.5
120	0.572	1.304	-0.161	0.54
121	1.966	3.075	0.856	0.51
122	9.471	13.376	5.566	0.23
123	2.16	3.312	1.008	0.5
124	3.498	4.515	2.48	0.51
125	0.457	1.454	-0.54	0.52
126	0.878	1.743	0.014	0.53
127	2.755	4.05	1.461	0.49
128	5.972	8.549	3.395	0.35
129	5.085	7.18	2.991	0.4
130	12.352	17.01	7.694	0.18
131	2.024	3.146	0.902	0.5
132	4.254	7.492	1.016	0.28
133	2.891	4.22	1.561	0.48
134	1.553	2.581	0.525	0.51
135	2.637	3.902	1.372	0.49
136	3.765	5.924	1.606	0.39
137	3.18	4.586	1.774	0.48
138	0.627	1.639	-0.384	0.51
139	1.147	2.022	0.271	0.53
140	3.305	5.541	1.069	0.38
141	4.591	6.854	2.328	0.38
142	0.163	1.145	-0.819	0.52
143	6.622	10.046	3.197	0.27
144	2.676	4.404	0.948	0.44
145	1.647	2.827	0.467	0.5
146	1.874	2.964	0.784	0.51
147	0.858	2.065	-0.349	0.5
148	3.1	4.678	1.522	0.46

149	2.065	3.58	0.549	0.46
150	2.743	3.91	1.576	0.5
151	2.282	3.228	1.335	0.52
152	3.257	4.395	2.119	0.5
153	1.663	3.055	0.27	0.48
154	10.098	15.917	4.28	0.13
155	1.337	2.787	-0.113	0.47
156	8.13	11.075	5.185	0.31
157	2.777	4.158	1.396	0.48
158	2.232	3.399	1.064	0.5
159	3.036	4.403	1.668	0.48
160	3.265	4.896	1.635	0.45
161	3.061	5.184	0.938	0.4
162	1.208	2.484	-0.068	0.49
163	4.074	5.733	2.415	0.45
164	25.776	34.734	16.819	0.07
165	24.099	32.479	15.719	0.07
166	10.373	14.066	6.68	0.25
167	21.276	28.685	13.866	0.09
168	10.156	16.007	4.306	0.13
169	3.707	5.482	1.932	0.43
170	3.856	5.68	2.031	0.43
171	1.872	3.104	0.639	0.49
172	1.315	2.615	0.014	0.49
173	1.533	2.688	0.378	0.5
174	0.914	1.846	-0.018	0.52
175	0.986	2.118	-0.147	0.5
176	5.859	9.384	2.335	0.26
177	2.249	3.578	0.92	0.48
178	3.95	8.085	-0.186	0.22
179	2.177	3.487	0.867	0.49
180	10.092	13.69	6.493	0.25
181	7.296	9.967	4.625	0.34
182	3.81	5.62	2.001	0.43
183	4.284	6.005	2.563	0.44
184	12.69	17.848	7.531	0.16
185	0.588	1.755	-0.58	0.5
186	1.767	3.356	0.178	0.46
187	4.863	6.366	3.361	0.47
188	91.714	143.363	40.066	0
189	21.312	41.953	0.671	0.01
190	2.061	3.191	0.931	0.5
191	3.36	4.672	2.048	0.49
192	1.473	2.516	0.43	0.51
193	1.102	1.972	0.232	0.53
194	1.458	2.469	0.446	0.51
195	5.714	8.196	3.232	0.36
196	-1.047	0.195	-2.289	0.49
197	1.356	2.35	0.362	0.52
198	1.647	2.596	0.698	0.52

199	5.457	7.537	3.376	0.4
200	15.915	21.488	10.342	0.14
201	8.354	11.373	5.335	0.3
202	10.849	14.702	6.996	0.23
203	6.399	10.208	2.589	0.24
204	0.108	1.089	-0.873	0.52
205	1.972	3.083	0.862	0.51
206	2.202	3.518	0.886	0.48
207	2.381	3.746	1.016	0.48
208	3.725	5.866	1.583	0.39
209	2.786	4.088	1.483	0.49
210	1.923	3.167	0.678	0.49
211	0.565	1.297	-0.167	0.54
212	3.058	4.432	1.685	0.48
213	8.845	12.508	5.181	0.25
214	2.877	4.203	1.551	0.48
215	2.752	3.64	1.865	0.53
216	18.449	25.876	11.021	0.09
217	1.538	2.491	0.586	0.52
218	2.93	4.456	1.405	0.46
219	2.954	4.384	1.524	0.47
220	3.13	4.611	1.65	0.47
221	0.742	2.219	-0.735	0.47
222	2.336	3.528	1.144	0.5
223	2.457	3.678	1.237	0.49
224	3.595	5.684	1.507	0.4
225	2.301	3.485	1.117	0.5
226	2.68	4.13	1.229	0.47
227	1.755	2.771	0.739	0.51
228	6.727	9.848	3.607	0.29
229	5.595	8.551	2.639	0.31
230	1.239	2.522	-0.044	0.49
231	1.838	3.063	0.614	0.49
232	5.727	11.484	-0.03	0.14
233	2.783	4.085	1.482	0.49
234	1.485	2.829	0.141	0.48
235	4.249	6.208	2.291	0.41
236	2.568	4.257	0.879	0.44
237	3.625	5.003	2.247	0.48
238	1.589	2.425	0.754	0.53
239	2.583	3.585	1.581	0.52
240	0.164	1.298	-0.971	0.5
241	6.794	10.814	2.773	0.22
242	4.707	7.633	1.781	0.31
243	1.408	2.879	-0.064	0.47
244	5.202	7.335	3.069	0.39
245	2.036	3.161	0.912	0.5
246	3.477	4.965	1.989	0.47
247	2.213	3.378	1.047	0.5
248	6.162	8.809	3.515	0.34

249	2.934	4.759	1.109	0.43
250	3.242	4.665	1.819	0.47
pooled	3.196	3.44	2.952	100

.
